# Timing of thoracic radiotherapy in the treatment of extensive-stage small-cell lung cancer: important or not?

**DOI:** 10.1186/s13014-017-0779-y

**Published:** 2017-02-28

**Authors:** Jing Luo, Liming Xu, Lujun Zhao, Yuanjie Cao, Qingsong Pang, Jun Wang, Zhiyong Yuan, Ping Wang

**Affiliations:** Department of Radiation Oncology, Tianjin Medical University Cancer Institute and Hospital, National Clinical Research Center for Cancer, Key Laboratory of Cancer Prevention and Therapy, Tianjin’s Clinical Research Center for Cancer, Tianjin, 300060 China

**Keywords:** Small cell lung cancer, Radiotherapy, Early/late radiotherapy, Prognosis

## Abstract

**Background:**

This study evaluated the prognosis of patients with extensive-stage small-cell lung cancer (ES-SCLC) that may be associated with timing of thoracic radiotherapy (TRT).

**Methods:**

ES-SCLC patients (*n* = 232) without progression were retrospectively analyzed after first-line induction chemotherapy. Patients in the TRT group were stratified as early-TRT (ERT; ≤3 cycles of induction chemotherapy received prior to TRT, *n* = 65) or late-TRT (LRT; >3 cycles, *n* = 122). To avoid selection bias, we conducted Propensity Score Matching (PSM) for patients. Overall survival (OS), progression-free survival (PFS), and locoregional recurrence-free survival (LRRFS) were assessed and compared.

**Results:**

Overall, the median survival time, PFS, and LRRFS were 13.2, 8.7, and 14.6 months, respectively. After matching by PSM, there were 45 patients total in the TRT/non-TRT groups, and 56 patients total in the ERT/LRT groups. OS, PFS, and LRRFS were significantly longer in the TRT group than the non-TRT group (*P* < 0.001, all). However, between the ERT and LRT groups these survival parameters were similar (*P* > 0.05, all).

**Conclusion:**

For ES-SCLC patients without progression, the addition of TRT after first-line chemotherapy benefited survival greatly. Early TRT showed no significant benefit over late TRT.

## Introduction

Small-cell lung cancer (SCLC) is well known for its aggressiveness and high propensity to metastasize [1, 2]. Although SCLC only accounts for 13–20% of all lung cancers [3], but it is still responsible for up to 40,000 deaths every year all over the world [4]. According to the new tumor-node-metastasis (TNM) version 7 staging system, Extensive-stage small-cell lung cancer (ES-SCLC) is defined as the IV stage small cell lung cancer and IIIB stage small cell lung caner which has metastatic nodes outside the thorax and cannot be safely encompassed within a tolerable radiotherapy treatment plan [5]. Patients with ES-SCLC always have a poor prognosis and are generally treated palliatively, although SCLC is highly sensitive to chemotherapy and radiation [6]. The current standard treatment for ES-SCLC is platinum-based first-line chemotherapy, which has remained unchanged over the past three decades [2]. Numerous studies were conducted to assess whether the addition of thoracic radiotherapy (TRT) to chemotherapy in the treatment of ES-SCLC was beneficial, although many of these studies have shown that TRT may improve patient outcomes significantly [7–10]. Studies of limited-stage SCLC (LS-SCLC) suggest that early TRT with concurrent chemoradiotherapy (CCRT) can improve survival [11–13], but such studies for ES-SCLC are lacking. In the present study, we retrospectively evaluated the efficacy and prognosis associated with the timing of TRT within the chemotherapy regime, in a large population of ES-SCLC patients.

## Materials and methods

### Study design

After approval by Human Investigation Committee of our cancer hospital, we identified all patients with ES-SCLC by thoracic computed tomography (CT) and positron emission tomography-computed tomography (PET-CT), confirmed and treated at our cancer hospital, between 1 January 2011, and 31 December 2015. All of them received first-line chemotherapy. We excluded all patients with incomplete medical records, who experienced progression after chemotherapy, or who were lost to follow-up.

In total, we collected data of 232 patients with ES-SCLC without progression after first-line induction chemotherapy. These patients were apportioned to a TRT treatment group and non-TRT treatment group. The TRT treatment group was further stratified according to the timing of TRT treatment, as early-TRT (ERT) or late-TRT (LRT). In addition, the electronic medical record was used to obtain clinical information regarding gender, age, KPS, and number of metastases. Both treatment factors and clinical factors were included in the survival analysis.

### Chemotherapy

Patients received etoposide + cisplatin/etoposide + carboplatin (EP/EC) first-line induction chemotherapy: etoposide 100 mg/m^2^ on days 1–5, combined with cisplatin 30 mg/m^2^ on days 1–3 or area under the curve (AUC)-based carboplatin dosing (AUC = 6). Every 21 days was a chemotherapy cycle. The median number of chemotherapy cycles was six.

### Radiotherapy

Radiotherapy planning was performed with a Philips Pinnacle^3^ treatment planning system (Philips Medical Systems, USA). For all patients, gross tumor volume was identified based on the CT image, and included the tumor and the metastatic lymph nodes, Clinical target volume was based on the gross tumor volume, but also included the primary tumor bed, and metastatic lymph nodes before chemotherapy. The distance from the margin of the gross tumor volume to the clinical target volume was 5 mm. The distance from the margin of the clinical target volume to the planning target volume was 5–10 mm. The radiation dose was 45–60 Gy in 15–30 fractions, 1.8–3 Gy per fraction, one fraction per day. There were 187 patients who received radiotherapy: 177 of them received intensity-modulated radiation therapy; 6 of them received 3D conformal radiotherapy; and 4 of them received stereotactic radiotherapy, the radiation dose was 45-60Gy in 8–15 fractions. Other than TRT, 70 patients received radiotherapy for metastatic sites, and the radiation dose was 30–45 Gy in 10–15 fractions, one fraction per day. There were 26 patients who received prophylactic cranial irradiation (PCI); all of them received PCI after TRT, the radiation dose of PCI was 25 Gy in 10 fractions [14], one fraction per day.

### Different timing of TRT

For analysis, the study population (*n* = 232) was divided into a TRT group (*n* = 187) and a non-TRT group (*n* = 45). Patients in the TRT group were stratified by the number of induction chemotherapy cycles they received prior to TRT. According to the previous research [15], we defined ERT as receiving TRT before or at the third cycle of chemotherapy, LRT was receiving TRT after the third cycle of chemotherapy. Specifically, the ERT group (≤3 cycles, *n* = 65) and the LRT group (>3 cycles, *n* = 122).

### Endpoint

Overall survival (OS) was estimated from the date of pathological diagnosis to the date of death for any reason or last follow-up. Progression-free survival (PFS) was from the date of receiving treatment to the date of disease progression or the date of death for any reason or last follow-up. Locoregional recurrence-free survival (LRRFS) was estimated from the date of receiving treatment to the date of local recurrence or the date of death for any reason or last follow-up.

### Criteria for evaluating therapeutic effect

The response rate was evaluated by chest CT scans 1 month after completion of TRT. In accordance with Response Evaluation Criteria in Solid Tumors version 1.1 (RECIST v. 1.1), short-term efficacy was considered a complete response, partial response, stable disease, or progressive disease. Immeasurable lesions (such as bone metastasis sites, or malignant pleural effusion) were generally not evaluated, unless involved in disease progression.

### Evaluation of therapy-associated toxicity

Toxic effects due to the therapy were assessed according to the National Cancer Institute Common Toxicity Criteria version 4. These included leukopenia, thrombocytopenia, anemia, nausea, and vomiting. Radiation-induced pneumonitis and esophagitis were also evaluated.

### Statistical analysis

Clinical characteristics were compared using the chi-squared test, Kaplan-Meier curves were created for OS, PFS, and LRRFS. The Cox proportional hazards model was used to examine factors associated with increased hazard of death. All statistical analyses were performed using SPSS 18.0 software (SPSS, Chicago, IL, USA) and R 2.8.0 statistical package. Because of the nonrandomized nature of this study, the ERT and LRT groups were matched for possible confounding variables: gender, age, KPS, smoking index, family history of neoplasm, weight loss, PCI and number of metastases ≥2, we preformed PSM with logistic regression estimation algorithm and nearest neighbor matching algorithm, and the caliper width was 0.2, matching 1:1. A group of 187 patients with RT was matched to 45 patients with non-RT, and another group of 122 patients with LRT was matched to 65 patients with ERT, based on the following baseline characteristics: age, gender, smoking index, weight loss, KPS, number of metastases, family history of neoplasm, and PCI *P* < 0.05 was considered statistically significant.

## Results

### Clinical characteristics

A total of 407 patients with pathologically diagnosed ES-SCLC were treated at our cancer hospital between 1 January 2011 and 31 December 2015. All of them received first-line chemotherapy. Ninety patients were excluded from the study because of disease progression after chemotherapy, and 85 were lost to follow-up. Finally, the present analysis included 232 patients. The clinical characteristics of the study cohort are listed in Table 1.

**Table 1:** Clinical characteristics and survival-related factors of 232 patients with ES-SCLC in univariate and multivariate analysis

		Univariate Analysis			Multivariate Analysis	
		Patients, n (%)	MST, mo	*P*	OR (95% CI)	*P*
Age, y	≥65	64 (27.6)	13.0	0.313		
	<65	168 (72.4)	13.8			
Gender	Male	182 (78.4)	12.4	0.027	0.798 (0.510–1.248)	0.323
	Female	50 (21.6)	19.3			
KPS	≥80	215 (92.7)	13.8	0.296		
	<80	17 (7.3)	9.7			
Smoking	Yes	188 (81.0)	12.8	0.191		
	No	44 (19.0)	16.3			
PCI	Yes	26 (11.2)	22.3	0.010	0.587 (0.340–1.015)	0.057
	No	206 (88.8)	12.1			
Distant metastasis	Yes	200 (86.2)	14.7	0.108		
	No	32 (13.8)	18.9			
Number of metastases ≥ 2	Yes	151 (65.1)	12.1	0.037	1.443 (0.860–2.422)	0.165
	No	81 (34.9)	15.8			
Weight loss	Yes	27 (11.6)	11.6	0.304		
	No	205 (88.4)	13.8			
TRT	Yes	187 (80.6)	15.0	<0.001	0.374 (0.262–0.532)	<0.001
	No	45 (19.4)	8.3			
TRT	ERT	65 (28.0)	14.6	0.720	1.185 (0.814–1.727)	0.376
	LRT	122 (52.6)	15.4			

### Univariate and multivariate cox proportional hazard analysis

Univariate analysis revealed that gender, PCI, number of metastases ≥ 2, and TRT were significantly important to OS. These significant variables were then put into a multivariate Cox proportional hazard analysis and the results showed that only TRT (odds ratio [OR], 0.374; 95% confidence interval [CI], 0.262–0.532, *P* < 0.001) retained its statistical significance (Table 1).

### Survival outcomes

For the entire study population, the median survival time was 13.2 months (95% CI 11.3–17.9 months); the OS rates at 6 and 12 months were 86.5 and 55.6%, respectively. The median PFS was 8.7 months (95% CI 7.6–9.7 months); the PFS rates at 6 and 12 months were 76.1 and 34.6%. The median LRRFS was 14.6 months (95% CI 11.6–14.8 months); the LRRFS rates at 6 and 12 months were 80.0 and 56.9%.

The patients of the TRT and non-TRT, ERT, and LRT groups were comparable with respect to gender, age, smoking index, family history of neoplasm, weight loss KPS, number of metastases ≥ 2, and PCI after propensity score matching (Table 2 and Table 3). Importantly, the characteristics listed in Tables 2 and Table 3 between the TRT and non-TRT, ERT, and LRT groups were not significantly different after PSM.

**Table 2:** Clinical characteristics of 232 patients in RT and non-RT groups after propensity score matching

		Before matching			After matching		
		RT^a^	non-RT^b^	*P*	RT^c^	non-RT^d^	*P*
Gender	Male	146	36	0.843	32	36	0.462
	Female	41	9		13	9	
Age, y	≥65	50	14	0.580	10	14	0.475
	<65	137	31		35	31	
KPS	≥80	174	41	0.749	42	41	1.000
	<80	13	4		3	4	
Smoking index	≥400	146	35	1.000	34	35	1.000
	<400	41	10		11	10	
Family history of neoplasm	Yes	39	8	0.836	14	8	0.220
	No	148	37		31	37	
Weight loss	Yes	25	2	0.121	4	2	0.677
	No	162	43		41	43	
PCI	Yes	25	1	0.034	2	1	1.000
	No	162	44		43	44	
Number of metastases ≥2	Yes	21	11	0.029	8	11	0.606
	No	166	34		37	34	

^a^ *n* = 187; ^b^ *n* = 45; ^c^ *n* = 45; ^d^ *n* = 45

**Table 3:** Clinical characteristics of 187 patients in the ERT and LRT groups after propensity score matching

		Before matching			After matching		
		ERT^a^	LRT^b^	*P*	ERT^c^	LRT^d^	*P*
Gender	Male	51	95	0.541	44	44	1.000
	Female	14	27		12	12	
Age, y	≥65	20	30	0.389	19	18	1.000
	<65	45	92		37	38	
KPS	≥80	62	112	0.548	53	54	1.000
	<80	3	10		3	2	
Smoking index	≥400	51	95	1.000	45	43	0.818
	<400	14	27		11	13	
Family history of neoplasm	Yes	8	31	0.039	8	9	1.000
	No	57	91		48	47	
Weight loss	Yes	6	19	0.265	6	6	1.000
	No	59	103		50	50	
PCI	Yes	15	10	0.006	7	8	1.000
	No	50	112		49	48	
Number of metastases ≥2	Yes	11	20	1.000	9	12	0.629
	NO	54	102		47	44	

^a^ *n* = 65; ^b^ *n* = 122; ^c^ *n* = 56; ^d^ *n* = 56

**Fig. 1:**
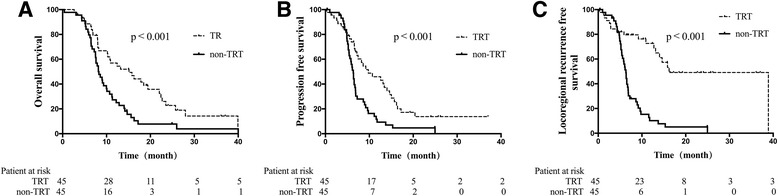
Survival rates of the TRT and non-TRT groups. Relative to patients in the non-TRT group, the survival rates of patients who received TRT showed improved (**a**) OS, (**b**) PFS, and (**c**) LRRFS

**Fig. 2:**
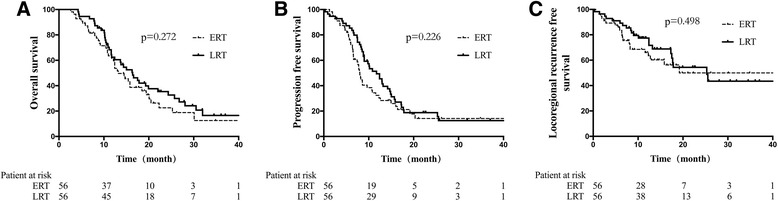
Survival rates of the ERT and LRT groups. The ERT and LRT groups were similar for (**a**) OS, (**b**) PFS, and (**c**) LRRFS

Relative to the non-TRT group, patients who received TRT experienced improved OS, (17.7 mo cf. 11.3 mo), PFS (9.8 mo cf. 6.3 mo), and LRRFS (16.2 mo cf. 6.3 mo; *P* < 0.001, all; Fig. 1, Table 4). The ERT and LRT groups were comparable in OS (16.4 mo cf. 14.5 mo; *P* = 0.272), PFS (8.0 mo cf. 11.7 mo; *P* = 0.226), and LRRFS (25.3 mo cf. 19.2 mo; *P* = 0.498; Fig. 2, Table 4).

**Table 4:** Survival rates of the TRT, non-TRT, ERT, and LRT groups

	OS		PFS		LRRFS	
	Overall, mo (95% CI)	1-year, %	Overall, mo (95% CI)	1-year, %	Overall, mo (95% CI)	1-year, %
TRT	17.7 (14.1-18.8)	57.9	9.8 (8.4–11.3)	45.9	16.2 (13.5–24.9)	72.6
Non-TRT	11.3 (9.8–12.7)	27.3	6.3 (5.0–7.0)	9.3	6.3 (5.6–7.0)	10.2
*P*	<0.001	–	<0.001	–	<0.001	–
ERT	16.4 (13.4–18.4)	66.2	8.0 (7.0–9.0)	34.2	25.3 (21.5–27.9)	69.8
LRT	14.5 (12.2–17.0)	60.5	11.7 (9.1–13.4)	44.1	19.2 (13.1–23.1)	63.8
*P*	0.272	–	0.226	–	0.498	–

Not only with regard to 3 cycles of induction chemotherapy, the OS, PFS and LRRFS of patients who received different cycles of induction chemotherapy were similar (Table 5). It seems that patients in the LRT group had better OS and PFS, and patients in the ERT group had better LRRFS; but the difference was not statistically significant.

**Table 5:** OS, PFS, and LRRFS in patients who received different cycles of induction chemotherapy before TRT

Cycles		OS, mo	*P*	PPFS, mo	*P*	LRRFS, mo	*P*
2	≤2	13.2	0.126	7.8	0.058	19.2	0.985
	>2	16.3		10.8		18.1	
3	≤3	16.4	0.272	8.0	0.226	25.3	0.498
	>3	14.5		11.7		19.2	
4	≤4	15.0	0.364	8.2	0.121	24.4	0.515
	>4	16.3		11.7		17.5	
5	≤5	14.7	0.253	8.2	0.176	24.4	0.338
	>5	16.3		11.8		17.5	
6	≤6	15.0	0.382	10.0	0.391	19.2	0.906
	>6	16.3		13.1		17.5	

### Toxic effects

Side effects were evaluated according to World Health Organization standards. In the present study, side effects of grade II and above (hematologic toxicity, gastrointestinal toxicity, acute radiation-induced pneumonitis, and esophagitis) were defined as toxic effects. The toxic effects of the TRT group were much greater than that of the non-TRT group (86.1 and 72.6%, *P* = 0.031). However the severe toxic effects (grade-III and above) of these groups were similar (33.6 and 29.2%, *P* = 0.105). In the TRT group, the incidence rates of acute radiation-induced pneumonitis or esophagitis were 9.1 and 11.8%, respectively. In the ERT and LER group, severe toxic effects were also similar (33.7 and 31.1%, *P* = 0.422).

## Discussion

This report from a single-institution study analyzed a cohort of patients with ES-SCLC who had not experienced progression after first-line induction chemotherapy. Our results show that TRT after induction chemotherapy could increase survival, reduce relapse, and improve local control in patients. Receiving ERT (with 3 or fewer chemotherapy cycles) was not associated with any difference in prognosis compared with LRT (more than 3 chemotherapy cycles).

For LS-SCLC, the role of TRT to improve survival is well established, but for ES-SCLC, the ability of TRT is still controversial [16]. At present, the basic treatment strategy for ES-SCLC patients rests on chemotherapy, in accordance with the old ACCP guideline, TRT can be considered after effective chemotherapy in selected patients [17, 18]. A well-known prospective randomized clinical trial has studied TRT in the treatment of ES-SCLC, Slotman et al. [7] demonstrated that TRT may improve patients’ outcomes significantly, but in their research, they failed to analyze the 1-year overall survival, and the radiation dose in the study was 30Gy in 10fractions. Comparing with Slotman’s research, our study improved the radiation dose, and we finally analyzed the 1-year OS, PFS and LRRFS. According to previous reports, more than half of ES-SCLC patients experienced thoracic failure after effective chemotherapy [19]. A prospective study in Canada, Yee et al. [20] demonstrated that TRT might increase the local control rate to prolong survival. There is also evidence that postoperative consolidative TRT for SCLC patients can greatly lower the local recurrence rate and therefore improve survival [21, 22]. In the present study, and we analyzed 112 patients after PSM, the OS, PFS, and LRRFS were all significantly better than that of patients who did not receive TRT (*P* < 0.01). In addition, rates of severe toxicity were not higher in the radiotherapy group.

SCLC is chemosensitive, therefore, effective chemotherapy can produce rapid responses with sometimes striking improvements in symptoms [3]. In the American College of Chest Physicians (ACCP) and National Comprehensive Cancer Network (NCCN) guidelines, ES-SCLC patients are advised to receive 4–6 cycles of first-line chemotherapy [18, 23]. Considering that LS-SCLC patients can benefit from early TRT after 2–3 cycles chemotherapy [24], is the timing of TRT in the treatment of ES-SCLC also important? The present study showed no evidence of a difference in OS, PFS, or LRRFS between patients who received early or late TRT. We conjecture that the difference may be associated with differences between LS- and ES-SCLC, the tumor of ES-SCLC is not entirely localized to the lungs, but always has distant organ metastases [8]. TRT is more powerful in improving local control than systemic control, and better local control surely can reduce the risk of distant metastases. However, for ES-SCLC, which always has distant metastases, the timing of TRT does not appear from the present study to affect the prognosis.

Although our results are applicable for patients with ES-SCLC, we recognize that there are limitations to the study. First, because of the retrospective nature of this analysis, there is unavoidable bias. Second, we acknowledge that differences in radiation dose, and methods of radiotherapy, second-line chemotherapy, and salvage radiotherapy may have contributed to study bias.

## Conclusion

In conclusion, after the start of first-line chemotherapy, TRT can prolong survival of patients with ES-SCLC without progression, and does not promote severe toxicity. Receiving TRT early or late does not significantly affect the prognosis, and the underlying mechanism is less clear. Further prospective studies are recommended.

## Abbreviations

CT: Computed tomography
EP/CE: Etoposide + cisplatin/etoposide + carboplatin
ERT: Early-TRT
ES-SCLC: Extensive-stage small-cell lung cancer
KPS: Karnofsky performance status
LRRFS: Locoregional recurrence-free survival
LRT: Late-TRT
LS-SCLC: Limited-stage small-cell lung cancer
OS: Overall survival
PCI: Prophylactic cranial irradiation
PFS: Progression-free survival
PSM: Propensity score matching
RECIST v. 1.1: Response evaluation criteria in solid tumors version 1.1
SCLC: Small-cell lung cancer
TRT: Thoracic radiotherapy
